# The Phytotoxic Potential of the Flowering Foliage of Gorse (*Ulex europaeus*) and Scotch Broom (*Cytisus scoparius*), as Pre-Emergent Weed Control in Maize in a Glasshouse Pot Experiment

**DOI:** 10.3390/plants9020203

**Published:** 2020-02-06

**Authors:** María Pardo-Muras, Carolina G. Puig, Pablo Souza-Alonso, Nuria Pedrol

**Affiliations:** 1 Department of Plant Biology and Soil Science, Faculty of Biology, University of Vigo, 36310 Vigo, Spain; mpardomuras@uvigo.es (M.P.-M.); cgpuig@uvigo.es (C.G.P.); pablo.souza@usc.es (P.S.-A.); 2 CITACA, Agri-Food Research, and Transfer Cluster, Campus da Auga, University of Vigo, 32004 Ourense, Spain; 3 Department of Soil Science and Agricultural Chemistry, University of Santiago de Compostela (USC), Escuela Politécnica Superior, 27002 Lugo, Spain

**Keywords:** allelochemicals, gorse, phytotoxicity, Scotch broom, soil microbial function, weed control, *Zea mays*

## Abstract

In our previous studies, the phytotoxicity of *Ulex europaeus* (gorse) and *Cytisus scoparius* (Scotch broom) was demonstrated in vitro and argued to be caused by the release of volatile and water-soluble compounds from fresh plant foliage. In light of these positive results, there was a need to test the effects *ex vitro*. In this work, gorse and Scotch broom were used as soil amendments in pot experiments in a glasshouse by incorporating slashed plant material into the soil at a ratio of 1% *w/w* on a dry mass basis. The phytotoxic effects on the emergence and early growth of maize and five accompanying weed species were analyzed, as were the effect on soil fertility and soil community-level physiological profiles. Thirty days after incorporation, significant decreases in weed density of 32.2% and 59.5% were found for gorse and Scotch broom soil amendments, respectively. Gorse soil amendment was notably effective impairing the establishment of *Amaranthus retroflexus* and diminishing the plant height of *Digitaria sanguinalis* and *Portulaca oleracea*. Scotch broom soil amendment was capable of significantly inhibiting the emergence of *D. sanguinalis*, *Convolvulus arvensis*, *P. oleracea*, and *A. retroflexus*, with a notable reduction of weed biomass. No undesirable side effects on maize crop or soil quality, including microbial activity, were detected. Our results suggest that the incorporation of gorse and Scotch broom foliage is promising for pre-emergent weed control in maize; however, field trials that support and expand these glasshouse results are essential.

## 1. Introduction

The use of allelopathic green manures is gaining attention as a complementary tool for integrated weed management [1,2,3,4,5,6]. Green manures are fast-growing crops which are cut and buried to improve soil properties by increasing nutrient retention, organic matter, and microbial biomass, while protecting the soil in between cash crops [7]. If an allelopathic crop is used for green manuring, when incorporated into the soil such a crop can release phytotoxic compounds capable of inhibiting the germination and growth of weeds in the soil seed bank. Different legume and cereal species have been reported to have suppressive effects on weeds [6,8,9,10,11]. Due to their richness in secondary bioactive metabolites, residues of certain medicinal plants have also been assayed as bioherbicidal green manures [12,13]. However, the use of green manures is quite a demanding practice, since growing a crop requires labor, suitable environmental conditions, seeds, nutrients, irrigation, and inputs for pest control [7]. Moreover, they take time and space in the crop schedule during their growth period, thus competing with cash crops.

The use of plant residues from allelopathic foliage available in the agroecosystem, instead of cultivated plants, has recently been considered to reduce the investment of time, costs and fieldwork aimed at green manure production. Some promising ‘biologically inspired’ studies endorse this idea. Puig et al. [14,15] have shown that the incorporation of *Eucalyptus globulus* leaves as a soil amendment inhibited the emergence of some common weeds of the maize, and notably reduced weed biomass throughout the whole growing period. Souza-Alonso et al. [16], using residues from *Acacia dealbata* and *A. longifolia* incorporated into the soil, have observed some negative effects on dicotyledon weeds. These practices sum up the recycling of waste material from the agroecosystem, that is, allelopathic forest residues and invasive species, and their use in eco-friendly practices for plant protection.

*Ulex europaeus* L. (gorse) and *Cytisus scoparius* (L.) Link. (Scotch broom) are two leguminous shrub species native to the Atlantic region. Gorse is native to the western coast of continental Europe and the British Isles, whereas Scotch broom is widely distributed all across Europe. In Galicia (NW Spain) and north Portugal, gorse and Scotch broom native shrubland cover 530,000 ha and 470,000 ha, respectively [17]. The populations of gorse and Scotch broom form dense canopies that provide large amounts of fresh biomass throughout the year, and thus have received increased attention as sources of inexpensive biomass [18]. Outside their natural distribution range, both legume shrubs are well-recognized as highly invasive weeds worldwide [19,20].

Pardo-Muras et al. [21,22,23] have studied the phytotoxicity of gorse and Scotch broom in vitro. The fresh foliage of both species could produce and release volatile organic compounds [21] and water-soluble compounds [22] capable of inhibiting the germination and early growth of the agricultural weeds *Amaranthus retroflexus* L. and *Digitaria sanguinalis* (L.) Scop, without affecting maize. Powerful synergistic effects among these allelopathic compounds were described [23] underlying the phytotoxicity on both weeds. These authors have shown that the flowers, as well as the vegetative parts, exerted different levels of phytotoxicity on different target weed species and physiological process (germination or early growth). Nevertheless, the most relevant bioherbicidal effects were described for the flowering foliage, coinciding with the highest amounts and richness of terpenoids, phenolic acids, and flavonoids from their chemical profiles. So, the use of gorse and Scotch broom flowering foliage as soil amendments could be a feasible way of profiting from the synergistic effects of their allelochemicals, acting together as a phytotoxic ‘cocktail’ capable of controlling weeds.

However, soil plays a crucial role in the fate of the bioactive compounds released by living plants or plant residues, so that many compounds that are effective in the laboratory could have little or no effectiveness in the field due to rapid degradation by microorganisms, and other biotic or abiotic interactions with soil [24,25]. Moreover, the allelochemicals released could produce undesirable side effects on crops, and/or change important parameters of soil fertility, including the metabolic activity of the edaphic microbial community [26,27,28].

To assess the efficiency of both species incorporated as soil amendment for pre-emergent weed control, as well as their possible side effects on maize and soil quality, the objectives of the present study were: (i) to evaluate in pot experiments the effects of the flowering foliage of gorse and Scotch broom on the establishment and early growth of maize and associated problematic weeds; and (ii) to study the potential side effects on soil fertility, including the functional capacity of the soil microbial community.

## 2. Results

### 2.1. Effects of Gorse and Scotch Broom Foliage Used as Soil Amendments on Weeds and Maize

**Figure 1:**
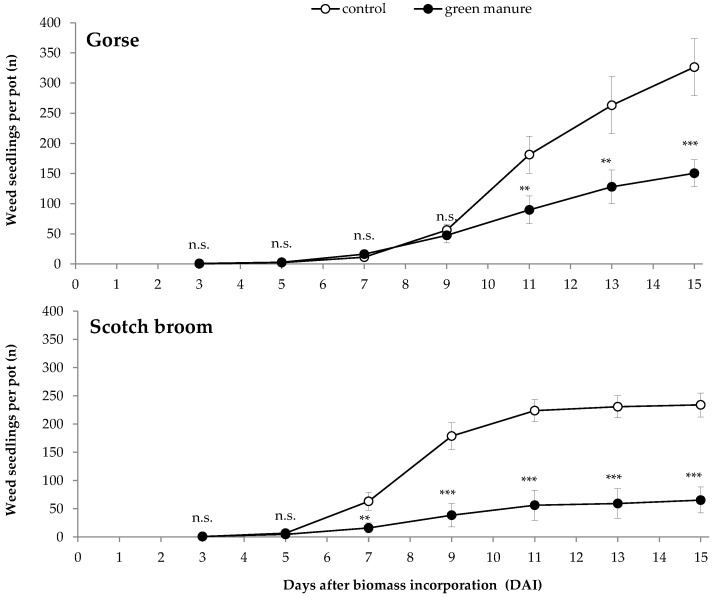
Weed emergence over 15 days after the incorporation of flowering foliage of gorse (above) or Scotch broom (below) into the soil as an amendment in two pot experiments. Symbols represent mean values of four replicates ± standard deviation (SD). Asterisks denote significant differences relative to the control ** *p* ≤ 0.01; *** *p* ≤ 0.001; n.s. not significant *p* > 0.05 (independent samples *t*-test).

Both species’ foliage incorporated as soil amendments conspicuously reduced the early weed emergence (Figure 1). For gorse, the effects on weed emergence were significant from 11 days after foliage incorporation (DAI), and the number of weed seedlings per pot on day 15 was reduced by 53.9% (*p* ≤ 0.001). For Scotch broom-amended pots, the effects were earlier and stronger than for gorse, with significant differences relative to control from 7 DAI, and 72.1% fewer weed seedlings than the control at 15 DAI (*p* ≤ 0.001). The gorse and the Scotch broom experiments were carried out with 20 days apart. The mean temperature during each assay was 11.7 °C and 12.8 °C for gorse and Scotch broom, respectively. Detailed temperatures during both experiments are provided as supplementary data (Figure S1).

Thirty days after the incorporation of the plant material (Table 1), gorse-amended pots showed a mean reduction of 32.2% of the total number of seedlings per pot relative to control, due to the significant inhibition of dicotyledon emergence (*p* ≤ 0.001). In sum, weed biomass was significantly diminished to 37.6% of control (*p* ≤ 0.05). *Amaranthus retroflexus* and the other spontaneous dicotyledons from the agricultural soil seed bank were the weeds most significantly affected in number, whereas for *Portulaca oleracea* L. and *D. sanguinalis* the phytotoxic effects were also measurable in plant height. In comparison, the monocotyledon *D. sanguinalis* had a larger number of plants emerge and increased aerial biomass relative to control (*p* ≤ 0.05).

Scotch broom incorporated into the soil was more effective in controlling weeds than gorse. The numbers of seedlings per pot, both for monocots and dicots, were reduced in a highly significant manner (59.5%, *p* ≤ 0.001), leading to a notable 54.0% (*p* ≤ 0.01) reduction of the total weed biomass, mainly due to a marked effect on dicotyledon biomass (*p* ≤ 0.001; Table 1). Except for *Solanum nigrum* L., which was unaffected by Scotch broom, the number of seeded dicot weeds emerging was considerably reduced (from 77.8% to 55.6% compared to control pots), as well as for other spontaneous weeds. Unlike gorse, Scotch broom also had adverse effects on the emergence of *D. sanguinalis* (36.9% reduction, *p* ≤ 0.001), but these effects were not significantly reflected in the final biomass per pot. Finally, *Convolvulus arvensis* L., *P. oleracea*, and particularly *A. retroflexus* and other dicots from the soil seed bank had significant biomass reductions, the scarce seedlings of the latter being significantly shorter than those in the control pots.

No variable measured in the maize at 30 DAI was negatively affected by the addition of plant foliage (Table 2). Instead, maize yield in the Scotch broom experiment was significantly increased (*p* ≤ 0.01), from 50.1% to 70.3% of the total harvested biomass in control and treated pots, respectively.

### 2.2. Effects on Soil Community-Level Physiological Profile

The agricultural soil used in the present experiment, with any of the amendments supplied, presented high levels of microorganism functional richness, diversity, and evenness (Table 3). Even so, the community of microorganisms from soils treated with gorse was significantly capable of using more substrates than that of control soils (richness mean value of 30 vs. 28, respectively; *p* ≤ 0.05, Table 3), was relatively more diverse (diversity mean value of 3.20 vs. 3.12, respectively; *p* ≤ 0.05) and attained higher levels of evenness (0.99 vs. 0.97, respectively; *p* ≤ 0.05). The same trend was observed for the soils amended with Scotch broom, although the increase in the number of C-substrates used relative to the corresponding control soils was not statistically significant.

**Figure 2:**
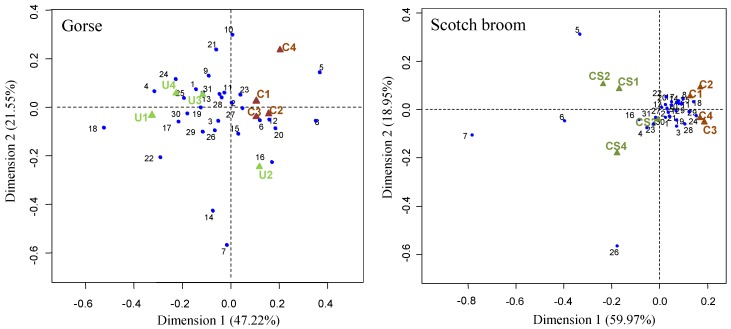
Correspondence analyses representing the bidimensional distribution of replicated soil microorganism analyses performed in control soils (C) and soils amended with gorse (U) or Scotch broom (CS) flowering foliage. C-substrates used by microorganisms in the BIOLOG Ecoplates are divided in six major classes: (a) carbohydrates, (b) carboxylic acids, (c) amino acids, (d) polymers, (e) amines/amides, and (f) phenolic compounds. From 1 to 31: 1. Pyruvic acid^(b)^; 2. Tween 40^(d)^; 3. Tween 80^(d)^; 4. *α*-cyclodextrin^(d)^; 5. Glycogen^(d)^; 6. Cellobiose^(a)^; 7. Lactose^(a)^; 8. *β*-methyl-D-glucoside^(a)^; 9. Xylose^(a)^; 10. Erythritol^(a)^; 11. Manitol^(a)^; 12. N-acetyl-D-glucosamine^(a)^; 13. D-glucosaminic acid^(b)^; 14. Glucose^(a)^; 15. D; L-*α*-Glicerol-P^(a)^; 16. D-Galactonic-*γ*-Lactone^(b)^; 17. D-Galacturonic acid^(b)^; 18. 2-Hydroxybenzoic acid^(f)^; 19. 4-Hydroxybenzoic acid^(f)^; 20. *α*-hydroxybutyric acid^(b)^; 21. Itaconic acid^(b)^; 22. *α*-ketobutyric acid^(b)^; 23. L-malic acid^(b)^; 24. L-arginine^(c)^; 25. L-asparagine^(c)^; 26. L-phenylalanine^(c)^; 27. L-serine^(c)^; 28. L-threonine^(c)^; 29. L-glutamic acid^(c)^; 30. Phenyletilamine^(e)^; 31. Putrescine^(e)^.

The plots obtained from the correspondence analysis are shown in Figure 2, where the C-sources preferred by microorganisms along with their corresponding samples are represented. The ordination plot showed some ordinated separation of samples, indicating some subtle preferences of C-source consumption depending on the soil treatment. For both experiments, two components accomplished the most variance (68.8% and 78.9% for gorse and Scotch broom, respectively). According to the C-substrate preference of soil microorganisms, in the case of the experiment with gorse foliage, the first dimension (accounting for 47.2% of the total variance; eigenvalue = 0.034) separated the control soils into different groups from the majority of soils supplied with gorse (Figure 2, left). However, the detailed separation criteria failed for one replication. In this dimension, most of the explained variance was represented by only two substrates: the polymer glycogen (label 5) and the carbohydrate *β*-methyl-D-glucoside (label 8), which grouped mostly with control soils. For the experiment with Scotch broom (Figure 2, right)**, the first dimension accounted for 60% of the variance (eigenvalue = 0.028), mainly dominated by the polymer glycogen (label 5), and the carbohydrates cellobiose (label 6) and lactose (label 7), the preference for which as C-sources for microorganisms separated the soils supplied with Scotch broom flowering foliage from the corresponding control soils.

### 2.3. Effects on Soil Physicochemical Parameters

All the physicochemical parameters measured were within the range of values for fertile agricultural soils, except the K/Mg balance, which was limiting for plant production in all cases (i.e., K/Mg > 0.5) (Table 4). After 30 days of plant growth, there were some significant differences between control soils (supplied with mineral and organic amendments), and those with the incorporation of shrub foliage (supplied with the mineral amendments only).

The soil amended with gorse showed significantly higher P contents and K/Mg balance values than the control pots. On the contrary, pH and the contents of the exchange cations Ca^2+^ and Mg^2+^ were significantly lower for the soil amendment, and the effective cation exchange capacity (CECe) also attained smaller values.

On the other hand, the soil amended with Scotch broom foliage maintained statistically equal levels of fertility than the corresponding control soil, and only the Ca/Mg balance significantly increased.

## 3. Discussion

The results obtained from the present glasshouse experiment showed the efficiency of the foliage of both species in reducing the emergence of weeds when used as soil amendments, while having no negative impact on maize establishment. After 30 days of foliage incorporation into the soil, decreases of weed density of 32.2% and 59.5% were measurable in the pots amended with gorse and Scotch broom, respectively, leading to early reductions of weed biomass of 37.6% and 54.0% without the use of any other method of weed control.

Gorse reduced the emergence of the dicot *A. retroflexus*, although neither its naturally emitted volatiles [21] nor its aqueous extract [22] have shown in vitro effects on germination. This successful control of germination under more realistic conditions could be due to the additive or interactive action of different allelochemicals [23]. It also could be possible that the seeds of *A. retroflexus* in the pot experiment germinated, but the early growth was impeded, and the seedlings did not manage to emerge. In contrast, *D. sanguinalis* was favored in gorse pots compared to control, possibly occupying the vacant space left by *A. retroflexus* and other dicots, as discussed by Álvarez-Iglesias et al. [6] for *Vicia faba* green manure. However, the seedlings of *D. sanguinalis* and those of the remaining dicots were shorter than the seedlings in control pots, which would reduce the intensity of competition for light. These results were consistent with the reductions in shoot length observed for *D. sanguinalis* and *A. retroflexus* in our previous in vitro studies with gorse, in which the benzenoid eugenol, the norisoprenoid theaspirane, and some phenolic compounds were argued to be involved [21,22].

Scotch broom flowering foliage used as a soil amendment significantly reduced the emergence of *C. arvensis* and *A. retroflexus*, and also significantly that of *P. oleracea* and the monocot *D. sanguinalis*. These species are among the most problematic agricultural weeds in European maize production [29]. Moreover, *C. arvensis* is a large seeded, fast-growing, and highly competitive weed with high tolerance to most pre-emergence herbicides [30]. Puig et al. [14] found that the incorporation of *E. globulus* leaves as a bioherbicidal amendment could control a wide range of common weeds, but was innocuous to *C. arvensis*. Therefore, the significant reduction of 77.8% of plants per pot of *C. arvensis*, with a final biomass reduction of 72.2%, was particularly notable.

Weed control exerted by Scotch broom in the pot experiments is consistent with our previous studies on its phytochemistry and phytotoxicity. In Pardo-Muras et al. [21], the volatiles emitted by the flowers of Scotch broom, particularly the oxygenated monoterpenes linalool, verbenone, and α-terpineol, have been shown to inhibit the germination of *A. retroflexus* and *D. sanguinalis*. Also, the growth of the surviving *D. sanguinalis* seedlings in pots could be impaired by the joint action of the oxygenated monoterpenes [23] together to water-soluble compounds (phenolic acids and flavonoids) [22].

Here, when the plant foliage was incorporated into the soil as an amendment, the phytotoxic effects on weeds were stronger than those in vitro in the absence of soil, reported in the studies previously cited [21,22]. The dose of foliage added to the soil in the current glasshouse approach was 1% *w/w* on a dry matter basis, in accordance with Puig et al. [14,15]. Such a quantity of foliage could emit VOCs into the soil pores enough to attain inhibitory concentrations, so being capable of reducing the germination and early growth of weeds. We suggest that the interactions among the VOCs emitted to the soil atmosphere, and also the water-soluble compounds released to the soil water, underlie the notable inhibition of the emergence and growth of weeds observed in the pot experiments. Such interactions among compounds of different chemical classes in the soil should be studied in more depth.

From the elemental analysis of our plant material, Scotch broom flowering foliage showed higher N contents than gorse (2.9% vs. 1.7%), resulting in a lower C: N ratio (16:1 vs. 27:1). This difference, together with the difference in mean temperature between both experiments (11.7 °C vs. 12.8 °C), may explain the earlier phytotoxic effects of Scotch broom (7 vs. 11 days after foliage incorporation if compared to gorse) because of the faster release of allelochemicals from less dense tissues. Dhima et al. [12], Kobayashi [24], and Kruidhof et al. [31] stated that the most significant release of allelochemicals into the soil is expected to occur in the early stage of the amendment decomposition, followed by an increase in nutrient availability. Recently, Puig et al. [32] argued that the release of volatile and water-soluble allelochemicals from *E. globulus* leaves was sustained after foliage incorporation. However, the duration was longer for the volatiles, which may last for more than one month, and even increase their release over time [32]. In our case, the phytotoxic effects on weeds were still evident 30 days after incorporation, particularly in the case of the volatile-rich flowering foliage of Scotch broom [21].

Unlike the effectiveness of gorse and Scotch broom for weed control, maize plants were unaffected. Large seeds like maize generally better tolerate the changes in soil chemical properties caused by green manures [33], and/or the effects of allelochemicals [31]. Moreover, in the pot experiments, the maximum phytotoxicity exerted on weeds at the initial physiological stages probably gave maize a competitive advantage. It is worth emphasizing that maize yields in pots supplied with Scotch broom soil amendment were increased relative to control pots. This effect could be due not only to the earlier and better weed control exerted, but also to the N content of Scotch broom tissues. In that sense, Hanifi and El Hadrami [34], and more recently Álvarez-Iglesias et al. [6], have pointed out that the supply of green manures with high N contents could mask the expectation of phytotoxic effects and facilitate the rapid growth of highly competitive species, which could be the case of *D. sanguinalis* when gorse was added to the soil. In our case, the use of BioF only in the control pots allowed us to distinguish the phytotoxic effects from the fertilizing effects of nitrogen of both leguminous species, and avoided an artificial inhibitory effect due to the limitation of nutrients in the control pots [6,35]. From our results, when used as soil amendments both species exerted phytotoxicity on weeds, and provided sufficient quantities of N for the healthy early growth of maize, as the BioF basal dressing did in the control pots. The shrub foliage may also supply other macronutrients, such as P, K^+^ and Ca^2+^. Substrates based on composted gorse foliage are currently marketed for gardening and horticulture, whereas Scotch broom plant material has been proposed as an optimum starting material for vermicomposting [36].

From the post-trial soil analysis, no fertility constraints caused by the incorporation of shrub foliage were detected. Some significant changes, in no case limiting for plant production, were identified in soils amended with gorse foliage. The detectable pH decrease could be due to the richness in phenolic compounds readily releasable to water [22]. The reductions in CECe and exchangeable cations Ca^2+^ and Mg^2+^ were very far from limiting [37]. However, in soils with low fertility levels, the effects on nutrient availability due to the incorporation of amendments should be carefully followed, especially for those with high C:N ratios for which nutrient immobilization by microorganisms is longer lasting. In our case, the addition of complete mineral amendments to both control and amended pots was useful to avoid side effects on nutrient availability that could mask the own effects on weed control [6]. For instance, except for the balance Ca/Mg, no significant modifications on soil properties were observed due to the incorporation of Scotch broom, so the weed suppression observed in our pot experiments must be attributed to the allelochemicals released from the buried plant material. On the other hand, the excess of K^+^ could compromise the medium to long-term availability of Mg^2+^, as reflected by the balance K/Mg > 0.5 in both control and amended pots, so K^+^ rich phosphorous amendments should be applied with caution to fertile soils.

The microbial activity of the soil is a determining factor of the phytotoxicity dynamics [38] since soil microorganisms can employ, degrade, or transform chemical compounds released into the environment [24,25,39]. Likewise, microbial activity may also be affected by the incorporation of phytotoxic plant residues into the soil. Based on the high functional diversity of the agricultural soil used in the pot experiments, the observed increases in the richness, diversity, and evenness indices suggest that soil amendments with gorse and Scotch broom foliage probably stimulated someway microbial activity, since heterotrophic soil metabolism was expanded. So, despite the apparent phytotoxic effects on weeds, no adverse effects were detected on the soil microorganisms, but, by contrast, the addition of shrub foliage to the soil produced the typical enhancement of microbial activities generally described for regular green manures and other organic amendments [7,40,41]. Part of the significant differences of microbial diversity relative to control soils could be due to the high N contents of the legume residues. Initially, the soil microorganisms could have consumed C-sources that are more readily available such as sugars [42,43]. As the decomposition of the plant material progressed, they could also have used part of the released allelochemicals as C-sources [44], thus being implied in the allelopathic processes by metabolizing, inactivating, or giving rise to other bioactive compounds [24].

Differential usages of C-sources were observed for gorse- and Scotch broom-amended soils if compared to control treatments, as indicated by the correspondence analysis separation. Recently, Li et al. [45] have confirmed that these changes are measurable using BIOLOG analysis at only one month after the applications of the amendment to the soil. For gorse, the preference for specific carbohydrate substrates like *β*-methyl-D-glucoside was more clearly linked to control soil samples, whereas the phenolic compounds (C-substrates 18 and 19) were represented close to the foliage-amended soils. This distribution may be related to the abundance of phenolics possibly released from gorse tissues to soil water. Samples of Scotch broom soils developed a preference for two disaccharides (C-substrates 6 and 7) in opposition to their corresponding control soils. In both cases, the proliferation of bacteria consuming these carbohydrates led to the increase of microorganisms able to metabolize the polymer glycogen (C-substrate 5). However, apart from these slight differences, the functional diversity and stability were very high in all of the soil samples, amended or not, with microbial richness capable of using from 27 to 31 out of 31 total C-sources.

The findings from our previous studies led us to test here, for the first time, the abundant foliage of gorse and Scotch broom incorporated into the soil for pre-emergent weed control in maize. Once their phytotoxic nature is unraveled [21,22,23], soil amendment with shrub foliage could be a feasible way of releasing a cocktail of allelochemicals of different chemical classes and modes of action. The cocktail may increase herbicidal efficiency and/or minimize the development of resistance. Moreover, the selective bioactivity on the weed species observed for gorse and Scotch broom soil amendment, as well as the specificity on a target weed or physiological process for different compounds [21], suggested that balanced mixtures of both species could be used to increase the bioherbicidal effect. In this sense, it is worth emphasizing that Pardo-Muras et al. [23] have shown intense synergistic interactions even between compounds of each species at quite low concentrations (in the range of few ppm).

The amounts of foliage added to soil in our experimental design (1% *w/w* on a dry mass basis in 5 L pots), corresponded to 11.1 t·ha^−1^ dw of gorse and Scotch broom. Such dosages are consistent with highly productive green manures, such as tall legumes and grasses (i.e., 10–15 t·ha^−1^ dw) [46]. The usage of the foliage of gorse and Scotch broom from the forest and invaded areas may provide the opportunity to transform and recycle the waste material of the forest industry and invasive species into raw materials for sustainable agriculture. This kind of multiple factor environmental approach will be in high demand in the new scenarios of weed management [47,48,49].

As a complementary tool in an integrated weed management strategy, the pre-emergent weed control of gorse and Scotch broom foliage amendments demonstrated in this paper may reduce the need for post-emergence control. The dependence on N-based fertilizers could also be reduced because being leguminous species. However, practical studies at field scale that support and expand on our glasshouse results are essential.

## 4. Materials and Methods

### 4.1. Soil and Plant Materials Description

Agricultural soil (A horizon) was collected from an agricultural field located in Vigo (Galicia, NW Spain, 42°12′15.6″ N, 8°46′19.8″ W). The field was dedicated to horticultural production for 15 years and then left fallow over the last two years. The soil was sieved through 2 mm mesh to remove debris and plant tissues. Soil physicochemical characteristics were pH (1:2.5 H_2_O) 6.2; EC < 0.16; organic matter 5.6%; total N 0.3%; concentrations of Ca^2+^, K^+^, Mg^2+^ and Na^+^, 7.9, 0.8, 1.22 and 0.14 cmol(+)·kg^−1^, respectively; and P^3−^ 82 mg·kg^−1^. Soil pH and electric conductivity (EC) were determined in a soil: water ratio of 1:2.5 (*w/v*). Organic matter was obtained by ignition in a muffle furnace (Carbolite ELF 11/14B) at 360 °C for 3 h. Total N and C contents were determined in a C-N analyzer (LECO CN-2000). Available P was extracted and measured according to Olsen et al. [50]. The cation exchange capacity (CEC) and exchangeable cation contents (Ca^2+^, Mg^2+^, K^+^) were determined according to Peech et al. [51]. The maximum water retention capacity (WRC) was 340 mL·kg^−1^ dw.

Flowering branches of gorse and Scotch broom were collected at different locations and dates, according to their respective full bloom. Gorse was collected in Cabo Home (Galicia, NW Spain, 42°16′08.9″ N, 8°51′38.0″ W) on 8 April 2016, while Scotch broom was collected in the vicinity of the University of Vigo (Galicia, NW Spain, 42°09′56.0″ N, 8°41′04.7″ W) on 27 April 2016. Samples of gorse (n. 75596) and Scotch broom (n. 75594) were deposited in the herbarium (SANT) at the University of Santiago de Compostela (USC). The dry weight/fresh weight ratios of the flowering branches were 0.33 and 0.31 for gorse and Scotch broom, respectively. Such ratios were obtained by drying sub-samples of fresh material at 60 °C until constant weight. Total C and N contents determined in triplicate (Fisons Instruments EA1108) were 46% and 1.7% for gorse, and 47% and 2.9% for Scotch broom, respectively, on a dry mass basis, Mean values of PO^4−^, K^+^, Mg^2+^ and Ca^2+^ determined by ICP-OES (Inductively coupled plasma - optical emission spectrometry) (Perkin Elmer Optima 4300DV) were 0.9, 2.5, 1 and 3.2 mg·g^−1^ for gorse, and 1.6, 2.3, 0.8 and 4.3 mg·g^−1^ for Scotch broom.

### 4.2. Soil Amendment with Shrub Foliage in Pot Experiments

Pot experiments were carried out under glasshouse conditions (natural light, T ≤ 26 °C maintained by a cooling system) according to the methodology used by Álvarez-Iglesias et al. [6] and Puig et al. [14]. Based on the full bloom dates for each shrub species, two independent glasshouse pot experiments were started 20 days apart (Figure S1). Plastic pots of 20 cm diameter (5 L) were filled with soil mixed with flowering fresh plant material slashed in 1 cm pieces at 1% dw/dw, corresponding to 106 or 113 g fw per pot for gorse or Scotch broom, respectively. These amounts were equivalent to 11.1 tons of dry weight per ha. The control treatment consisted of agricultural soil mixed with polypropylene drinking straws (1 cm length, 5 mm diam.) to mimic the padding effect of the same volume of plant material incorporated into the soil [52]. Mineral amendments were added at a basal dressing dose for maize: Patent PK (K+S KALI GmbH Kassel, Germany) [P_2_O_5_ 12%, K_2_O 15%, MgO 5%] at 800 kg·ha^−1^, and Calcimag granulated (Fertimón Calcimag, Spain) [CaO 36%, MgO 2.5%] at 3000 kg·ha^−1^. As is recommended practice in allelopathy studies [6,35], the control pots were supplemented with the organic amendment BioF (Aviporto, Spain) [N 3%, P_2_O_5_ 3%, K_2_O 3%] at 5682 kg ha^−1^, in order to make up for the nitrogen supplied by the N-rich plant tissues. In this way, the phytotoxic effects can be distinguished from the fertilizing effects. Each treatment was replicated four times. Pots were watered to the maximum WRC and weighed. Then, each pot was sown with five equidistant seeds of maize and five seeds of *C. arvensis* (field bindweed) at 2 cm depth. Moreover, 24 mg of seeds of all of the following species: *A. retroflexus* (redroot pigweed), *P. oleracea* (common purslane), *S. nigrum* (black nightshade), and *D. sanguinalis* (large crabgrass) were surface spread and evenly buried. Thus, the seed bank weed densities of infested corn fields were simulated (6 g·m^−2^; [12]). Seeds of maize cv. ‘Anjou 387’ were supplied by Limagrain Ibérica S.A. (Elorz, Spain). Seeds from all weeds were purchased from Herbiseed (Twyford, UK).

#### 4.2.1. Assessing the Effects of Gorse and Scotch Broom Foliage on Weeds and Maize

Weed and maize emergence were recorded daily until the control pots were crowded (day 15 after incorporation). Every two days, pots were relocated and weighed, and the water lost by evapotranspiration (ET) was replaced. Thirty days after foliage incorporation, the final number of seedlings was counted. Weeds were harvested by cutting at soil level, identified and separated by species. After measuring the plant height, each species was dried at 70 °C for 72 h to obtain the aerial biomass (g dw). Maize plants were processed as described for weeds, and their roots were also harvested, measured, and dried. V3 leaf was measured for the leaf area (CI-202 leaf area meter, CID Bio-Science, Inc., Camas, WA, USA), and then dried and weighed to obtain the Specific Leaf Area (SLA, m^2^·kg^−1^ dw). Maize yields were calculated as yield (%) = [maize aerial biomass/(maize aerial biomass + total weed aerial biomass)] × 100, as the percentage of the total harvested biomass corresponding to maize [6,14]. For each pot, relative water use efficiency was calculated as rWUE (g·L^−1^) = maize aerial biomass/total ET.

#### 4.2.2. BIOLOG Ecoplates for Soil Community-Level Physiological Profile (CLPP)

For the analyses of soil CLPP, 2 g of fresh soil from the rhizosphere of maize plants from each pot was diluted with 25 mL of NaCl (0.85%) in Falcon tubes (50 mL) and vortexed for 2 min. Suspensions were settled for 2–3 min and diluted to 10^−3^ [20]. The assessment of the CLPP was carried out using BIOLOG Ecoplate (Biolog Inc., Hayward, CA, USA) 96-well plates, containing 31 C-sources divided into six major groups: carbohydrates, carboxylic acids, amino acids, polymers, amines, and phenolic compounds. Each Ecoplate provided three measurement replications per pot. Each well was inoculated with 150 μL of the soil suspension. Ecoplates were maintained at 23 °C in the dark, and the evolution of the average well-color development (AWCD) was measured on SPECTRO star Nano-BMG Labtech at 595 nm after 24, 48, 72, 96, 120, 144 and 168 of incubation hours. In each Ecoplate, microbial response based on C-substrate consumption was calculated following the equation AWCD = ∑(C–R)/n*i*, where C is the color production for each well, R is the absorbance value of the control well to correct for background color, and n*i* is the number of substrates [53]. Relative rates of color production among samples were compared based on similar AWCD values as Garland [54], who noted that comparisons should be preferably made based on reference points comprised between 0.7 and 1 units of absorbance. In our case, to allow comparisons, mean values of AWCD comprised between 0.65–0.75 (gorse) and 0.7–0.9 (Scotch broom) were selected for each comparison. Wells that had negative values were set to zero for the analyses. The functional diversity of the microbial communities was estimated using three parameters: species richness, diversity, and evenness, as described by Zak et al. [55].

#### 4.2.3. Measuring Post-Trial Soil Physicochemical Parameters

After harvesting, soil from each control and foliage-amended pot was air-dried and sieved through 2 mm mesh for physicochemical analysis. Soil physicochemical parameters were analyzed according to the methodology described in Section 4.1.

### 4.3. Statistical Analyses

The experiment was conducted according to a completely randomized design. First, data were tested for normality using the Kolmogorov-Smirnov test, and non-normal values were log-transformed. The homogeneity of variances was analyzed by Levene’s test. Data were compared between treatments by independent samples *t*-test. These statistical analyses were carried out using the SPSS v.19 (IBM SPSS Inc., Chicago, IL, USA) software package for Windows. In the case of CLPP data, correspondence analysis (CA) was performed on normalized data for each well by using R statistical software (version 3.2.2, R Core Team, R Foundation for Statistical Computing, Vienna, Austria). Correspondence analysis plots were constructed for representing the C-sources preferred by microorganisms along with their corresponding samples (four replications per treatment) occurring in the BIOLOG Ecoplates, this is, the ordination space.

## 5. Conclusions

The results from pot experiments under semi-controlled glasshouse conditions provided positive evidence that gorse and Scotch broom foliage, incorporated into the soil as amendments at 1% (dw/dw), were effective for pre-emergent weed control. Significant reductions in the density and biomass of *Amaranthus retroflexus, Digitaria sanguinalis, Portulaca oleracea*, and *Convolvulus arvensis* were observed over 30 days. The weed suppression could be due to the release of cocktails of allelochemicals into the soil environment, from which volatile and water-soluble compounds may act together, additive or synergistically, on the weed seed bank. Concomitantly, no undesirable side effects on maize crops or soil quality were detected; otherwise, both species provided enough N quantities for maize basal dressing.

So, our results suggest that gorse and Scotch broom are promising sources of foliage with bioherbicidal potential. Their use as soil amendments can sum a new complementary tool in global eco-friendly weed management strategies. However, field trials that support and expand on our glasshouse results are essential.

## Figures and Tables

**Table 1:** General and specific effects of gorse or Scotch broom flowering foliage incorporated into the soil as amendments, on the establishment and growth of weeds 30 days after foliage incorporation. Values are the mean of four replicates ± SD.

	Control	Gorse	Sig.		Control	Scotch Broom	Sig.
**General effects on weeds**							
Seedlings per pot (n)	322.00 ± 23.48	218.25 ± 19.77	− − −		191.50 ± 18.41	77.50 ± 14.75	− − −
Dicotyledon seedlings per pot (n)	289.00 ± 23.85	169.25 ± 8.14	− − −		158.25 ± 19.45	56.50 ± 13.40	− − −
Monocotyledon seedlings per pot (n)	33.00 ± 2.83	49.00 ± 11.80	+		33.25 ± 2.22	21.00 ± 2.45	− − −
Weed biomass (mg)	1968.63 ± 384.93	1228.58 ± 294.21	−		2140.40 ± 377.39	984.93 ± 279.93	− −
Dicotyledons biomass (mg)	1917.38 ± 369.47	1119.18 ± 266.93	−		1913.55 ±352.90	557.83 ± 230.32	− − −
Monocotyledons biomass (mg)	51.25 ± 17.88	109.40 ± 47.87	n.s.		226.85 ± 67.08	427.10 ± 455.06	n.s.
							
**Effects on weed species**							
Plants per pot (n)							
*Convolvulus arvensis*	1.25 ± 0.96	2.00 ± 0.82	n.s.		2.25 ± 0.96	0.50 ± 0.58	−
*Amaranthus retroflexus*	113.25 ± 6.85	77.50 ± 6.81	− − −		80.25 ± 14.22	22.75 ± 8.42	− − −
*Solanum nigrum*	11.75 ± 4.72	10.50 ± 1.73	n.s.		10.00 ± 2.16	11.00 ± 4.55	n.s.
*Portulaca oleracea*	9.25 ± 2.50	11.50 ± 3.70	n.s.		6.75 ± 0.96	3.00 ± 1.63	− −
*Digitaria sanguinalis*	33.00 ± 2.83	48.75 ± 11.32	+		32.50 ± 2.65	20.50 ± 3.32	− − −
Other dicotyledons	153.50 ± 23.10	67.75 ± 3.50	− −		59.00 ± 6.68	19.25 ± 5.32	− − −
Other monocotyledons	0.00 ± 0.00	0.25 ± 0.50	n.s.		0.75 ± 0.96	0.50 ± 1.00	n.s.
							
Plant height (cm)							
*Convolvulus arvensis*	8.48 ± 6.57	6.50 ± 1.00	n.s.		11.35 ± 1.69	5.25 ± 6.10	n.s.
*Amaranthus retroflexus*	10.38 ± 0.64	7.70 ± 0.69	− − −		11.65 ± 0.31	7.55 ± 1.67	− − −
*Solanum nigrum*	5.59 ± 1.78	4.78 ± 0.64	n.s.		7.48 ± 1.11	7.93 ± 1.03	n.s.
*Portulaca oleracea*	1.87 ± 0.20	1.34 ± 0.23	−		1.78 ± 0.23	1.48 ± 0.47	n.s.
*Digitaria sanguinalis*	7.28 ± 0.84	5.93 ± 0.38	−		13.10 ± 1.75	11.00 ± 1.89	n.s.
Other dicotyledons	16.43 ± 2.16	12.63 ± 0.82	−		15.98 ± 1.00	11.68 ± 2.76	−
Other monocotyledons	0.00 ± 0.00	7.13 ± 14.25	n.s.		11.70 ± 21.57	11.03 ± 22.05	n.s.
							
Aerial biomass per pot (mg)							
*Convolvulus arvensis*	15.90 ± 12.04	17.55 ± 6.80	n.s.		38.27 ± 16.68	10.65 ± 12.31	−
*Amaranthus retroflexus*	370.75 ± 109.78	238.02 ± 71.74	n.s.		665.97 ± 109.50	137.80 ± 90.32	− − −
*Solanum nigrum*	30.30 ± 16.21	34.37 ± 8.50	n.s.		52.00 ± 12.68	128.37 ± 68.99	n.s.
*Portulaca oleracea*	1.82 ± 1.02	4.37 ± 1.87	n.s.		3.42 ± 0.95	1.82 ± 0.87	−
*Digitaria sanguinalis*	51.25 ± 17.88	93.77 ± 20.74	+		194.67 ± 61.60	142.25 ± 51.89	n.s.
Other dicotyledons	1489.60 ± 305.89	824.85 ± 199.03	− −		1.15 ± 0.26	0.28 ± 0.14	− − −
Other monocotyledons	0.00 ± 0.00	15.62 ± 31.25	n.s.		32.17 ± 64.21	36.90 ± 73.80	n.s.

Sig.: signs denote significant decrease (−) or increase (+) relative to the control: one sign, *p* ≤ 0.05; two signs, *p* ≤ 0.01; three signs, *p* ≤ 0.001; n.s., not significant (independent samples *t*-test).

**Table 2:** Effects of gorse or Scotch broom flowering foliage incorporated into the soil as amendments on different variables measured on maize 30 days after incorporation. Values are the mean of four replicates ± SD.

Variable	Control	Gorse	Sig.		Control	Scotch Broom	Sig.
Maize seedlings per pot (n)	2.00 ± 1.41	2.75 ± 1.26	n.s.		3.50 ± 0.58	3.67 ± 1.16	n.s.
Root length (cm)	31.79 ± 4.42	31.68 ± 6.17	n.s.		46.88 ± 7.03	46.03 ± 2.67	n.s.
Plant height (cm)	43.49 ± 2.58	40.74 ± 4.69	n.s.		59.85 ± 2.56	57.09 ± 1.98	n.s.
Root biomass per plant (g)	0.74 ± 0.04	0.85 ± 0.12	n.s.		0.95 ± 0.06	1.08 ± 0.16	n.s.
Aerial biomass per plant (g)	0.54 ± 0.09	0.48 ± 0.13	n.s.		1.00 ± 0.15	1.09 ± 0.24	n.s.
Total biomass per plant (g)	1.29 ± 0.13	1.33 ± 0.23	n.s.		1.95 ± 0.19	2.17 ± 0.39	n.s.
SLA (m^2^ kg^−1^)	56.66 ± 2.71	60.50 ± 3.56	n.s.		59.26 ± 6.04	56.26 ± 1.58	n.s.
Leaf area (cm^2^)	34.93 ± 5.55	29.29 ± 4.72	n.s.		40.03 ± 2.82	47.52 ± 7.59	n.s.
Maize yield (% of total harvest)	50.17 ± 6.15	58.11 ± 11.42	n.s.		50.10 ± 7.58	70.28 ± 5.25	+ +
Shoot: root ratio	0.73 ± 0.09	0.56 ± 0.11	−		1.06 ± 0.13	1.01 ± 0.12	n.s.
rWUE (g L^−1^)	0.34 ± 0.06	0.30 ± 0.10	n.s.		0.57 ± 0.09	0.59 ± 0.13	n.s.

SLA, specific leaf area. rWUE, relative water use efficiency. Sig.: signs denote significant decrease (−) or increase (+) relative to the control: one sign, *p* ≤ 0.05; two signs, *p* ≤ 0.01; n.s., not significant *p* > 0.05 (independent samples *t*-test).

**Table 3:** Effects of gorse or Scotch broom flowering foliage incorporated into the soil as amendments on soil microorganism substrate richness, diversity, and evenness. Values are the mean of four replicates ± SD.

	Gorse			Scotch Broom		
	Control Soil	Soil Amendment	Sig.	Control Soil	Soil Amendment	Sig.
Richness	28.00 ± 1.41	30.00 ± 0.82	+	29.50 ± 1.29	30.75 ± 0.50	n.s.
Diversity	3.12 ± 0.05	3.20 ± 0.02	+	3.25 ± 0.03	3.30 ± 0.02	+
Evenness	0.97 ± 0.01	0.99 ± 0.01	+	0.98 ± 0.01	0.99 ± 0.01	+

Sig.: signs denote significant increase (+) relative to the control: *p* ≤ 0.05; n.s., not significant *p* > 0.05 (independent samples *t*-test).

**Table 4:** Effects of gorse or Scotch broom flowering foliage incorporated into the soil as amendments on soil physicochemical parameters measured 30 days after foliage incorporation. Values are the mean of four replicates ± SD.

Variable	Control	Gorse	Sig.		Control	Scotch broom	Sig.
pH	6.86 ± 0.03	6.70 ± 0.08	−		6.86 ± 0.03	6.73 ± 0.29	n.s.
Soil EC (dS m^−1^)	0.15 ± 0.03	0.16 ± 0.02	n.s.		0.13 ± 0.03	0.15 ± 0.02	n.s.
Organic matter (%)	5.70 ± 0.12	5.58 ± 0.33	n.s.		5.25 ± 0.47	5.63 ± 0.05	n.s.
Total N (%)	0.29 ± 0.02	0.30 ± 0.02	n.s.		0.30 ± 0.05	0.28 ± 0.04	n.s.
Total C (%)	3.27 ± 0.15	3.38 ± 0.23	n.s.		3.32 ± 0.59	3.10 ± 0.35	n.s.
P (mg kg^−1^)	63.75 ± 1.89	68.00 ± 5.58	+		67.50 ± 5.26	65.00 ± 4.83	n.s.
Ca/Mg	5.50 ± 0.58	5.00 ± 0.00	n.s.		5.25 ± 0.50	6.25 ± 0.50	+
K/Mg	0.60 ± 0.00	0.70 ± 0.08	+		0.63 ± 0.05	0.73 ± 0.10	n.s.
CECe	12.72 ± 0.76	10.80 ± 0.26	− −		12.09 ± 0.91	12.27 ± 2.22	n.s.
*Exchangeable cations* (cmol _(+)_ kg^−1^):							
Ca^2+^	9.58 ± 0.67	7.58 ± 0.15	− − −		9.00 ± 0.78	9.25 ± 2.05	n.s.
Mg^2+^	1.72 ± 0.07	1.60 ± 0.04	−		1.66 ± 0.07	1.55 ± 0.17	n.s.
K^+^	1.02 ± 0.06	1.12 ± 0.10	n.s.		1.04 ± 0.06	1.11 ± 0.11	n.s.

CECe, effective cation exchange capacity. Sig.: signs denote significant decrease (−) or increase (+) relative to the control: one sign, *p* ≤ 0.05; two signs, *p* ≤ 0.01; three signs, *p* ≤ 0.001; n.s., not significant *p* > 0.05 (independent samples *t*-test). Underlined values are limiting for plant production.

## Supplementary Materials

The following are available online at https://www.mdpi.com/2223-7747/9/2/203/s1, Figure S1: Mean, maximum, and minimum daily temperature during the glasshouse pot experiments with flowering foliage of gorse or Scotch broom for pre-emergent weed control.
