# Supplementary material for: The Phytotoxic Potential of the Flowering Foliage of Gorse (Ulex europaeus) and Scotch Broom (Cytisus scoparius), as Pre-Emergent Weed Control in Maize in a Glasshouse Pot Experiment
Source: Plants (Basel). 2020 Feb 6;9(2):203. doi: 10.3390/plants9020203 (PMC7076520; doi:10.3390/plants9020203)
Supplement: Supplementary file 1 [file plants-09-00203-s001.pdf]

The Phytotoxic Potential of the Flowering Foliage of Gorse (*Ulex europaeus*) and Scotch Broom (*Cytisus scoparius*), as Pre-Emergent Weed Control in Maize in a Glasshouse Pot Experiment

María Pardo-Muras [1,2], Carolina G. Puig [1,2], Pablo Souza-Alonso [1,3] and Nuria Pedrol [1,2,*]

[1] Department of Plant Biology and Soil Science, Faculty of Biology, University of Vigo, 36310 Vigo, Spain; mpardomuras@uvigo.es (M.P.-M.); cgpuig@uvigo.es (C.G.P.); pablo.souza@usc.es (P.S.-A.)

[2] CITACA, Agri-Food Research, and Transfer Cluster, Campus da Auga, University of Vigo, 32004 Ourense, Spain

[3] Department of Soil Science and Agricultural Chemistry, University of Santiago de Compostela (USC), Escuela Politécnica Superior, 27002 Lugo, Spain

* Correspondence: pedrol@uvigo.es; Tel.: +34-986-812-616

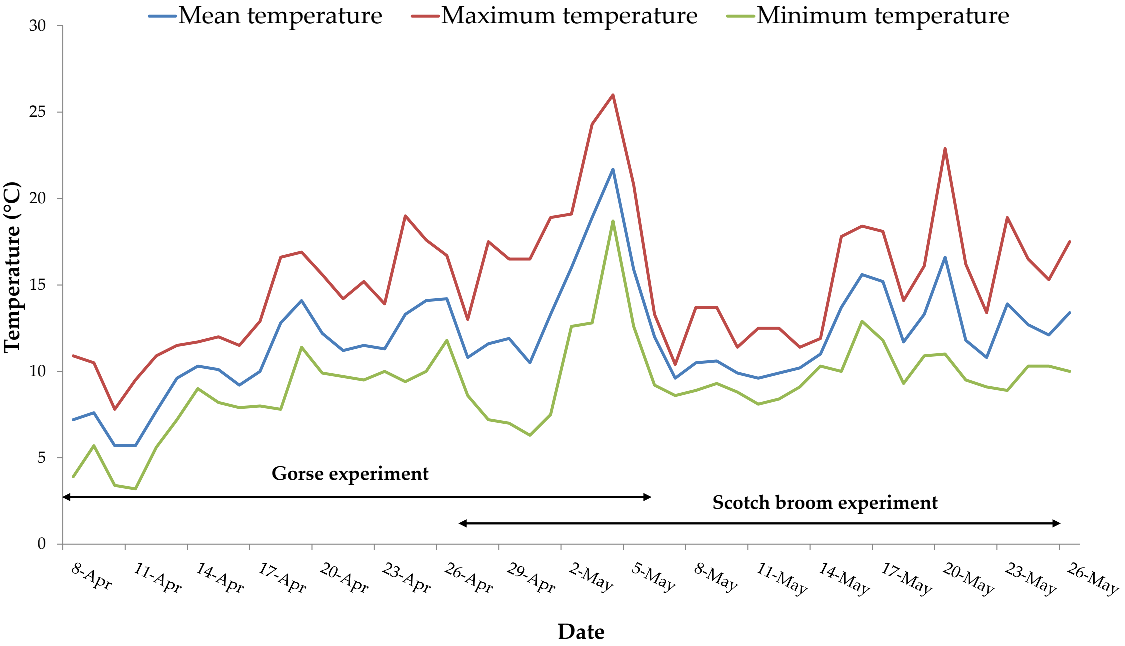

Figure S1. Mean, maximum, and minimum daily temperature during the glasshouse pot experiments with flowering foliage of gorse or Scotch broom for pre-emergent weed control.
